# Visualizing Interactions along the *Escherichia coli* Twin-Arginine Translocation Pathway Using Protein Fragment Complementation

**DOI:** 10.1371/journal.pone.0009225

**Published:** 2010-02-16

**Authors:** Jan S. Kostecki, Haiming Li, Raymond J. Turner, Matthew P. DeLisa

**Affiliations:** 1 Department of Biomedical Engineering, Cornell University, Ithaca, New York, United States of America; 2 Department of Biological Sciences, University of Calgary, Calgary, Alberta, Canada; 3 School of Chemical and Biomolecular Engineering, Cornell University, Ithaca, New York, United States of America; Auburn University, United States of America

## Abstract

The twin-arginine translocation (Tat) pathway is well known for its ability to export fully folded substrate proteins out of the cytoplasm of Gram-negative and Gram-positive bacteria. Studies of this mechanism in *Escherichia coli* have identified numerous transient protein-protein interactions that guide export-competent proteins through the Tat pathway. To visualize these interactions, we have adapted bimolecular fluorescence complementation (BiFC) to detect protein-protein interactions along the Tat pathway of living cells. Fragments of the yellow fluorescent protein (YFP) were fused to soluble and transmembrane factors that participate in the translocation process including Tat substrates, Tat-specific proofreading chaperones and the integral membrane proteins TatABC that form the translocase. Fluorescence analysis of these YFP chimeras revealed a wide range of interactions such as the one between the Tat substrate dimethyl sulfoxide reductase (DmsA) and its dedicated proofreading chaperone DmsD. In addition, BiFC analysis illuminated homo- and hetero-oligomeric complexes of the TatA, TatB and TatC integral membrane proteins that were consistent with the current model of translocase assembly. In the case of TatBC assemblies, we provide the first evidence that these complexes are co-localized at the cell poles. Finally, we used this BiFC approach to capture interactions between the putative Tat receptor complex formed by TatBC and the DmsA substrate or its dedicated chaperone DmsD. Our results demonstrate that BiFC is a powerful approach for studying cytoplasmic and inner membrane interactions underlying bacterial secretory pathways.

## Introduction

The bulk of protein transport across the inner membrane of Gram-negative bacteria occurs via the well-characterized Sec export pathway [1]–[4]. Sec export involves the membrane translocation of polypeptides that are largely unfolded and effectively ratchet their way through the Sec pore in a process requiring ATP hydrolysis [5], [6]. A fundamentally different pathway known as the twin-arginine translocation (Tat) system operates alongside the Sec pathway. The hallmark of the Tat pathway that distinguishes it from the Sec mechanism is the ability to transport proteins of varying dimension that have acquired a largely, if not completely, folded conformation [7]–[10]. Studies on the Tat mechanism have demonstrated that the integral membrane proteins TatA, TatB, and TatC form the minimal components necessary for exporting folded proteins in *E. coli*. The TatA and TatB components are single-span integral membrane proteins while TatC has been shown to contain six transmembrane spans [11]. These membrane proteins have been observed to form two distinct complexes: one that is comprised of multiple subunits of TatA and a second that contains predominantly TatB and TatC [12]–[14]. TatA homo-oligomers form a variable diameter ring structure that may serve as a protein-conducting channel [15] or a patch that facilitates translocation by local destabilization of the bilayer [16]. The TatB and TatC proteins form a complex to which substrates initially bind [17], suggesting that TatBC serves as the twin-arginine signal peptide binding site.

Proteins that transit the Tat pathway do so because they fold too rapidly to remain competent for Sec-dependent export [18] or because they bind protein subunits [9], [19] and/or redox cofactors [20], such as FeS clusters or molybdopterin centers, in the cytoplasm. This raises the important question of how the Tat pathway determines whether a substrate is sufficiently folded, including the assembly of subunits or cofactors, prior to the membrane translocation step. At least three mechanisms operate prior to, or concomitant with, translocation through the Tat pore that serve to prevent wasteful or harmful export of premature or improperly folded substrates. First, a folding quality control mechanism has been proposed on the basis that misfolded or partially folded proteins are not exported via the Tat pathway [7], [21]–[23]. Recent evidence suggests that the Tat translocase itself apparently “senses” the substrate folded state [24]. Second, Tat export is regulated at an earlier stage by additional “proofreading” factors that recognize specific Tat signal peptides and/or mature domains. These factors include dedicated chaperones such as DmsD and TorD that coordinate the cofactor-insertion and export processes [25], [26] and general molecular chaperones (e.g., DnaK, SlyD) that affect the stability and targeting of certain substrates [26]–[28]. Third, the Tat apparatus appears to directly initiate the turnover of rejected substrate molecules [29].

Direct visualization of the molecular interactions between proteins can reveal important details about how protein-protein interactions execute and regulate a wide range of events inside living cells. A number of fluorescence-based methods have been developed and widely used for visualizing and identifying interacting proteins including fluorescence resonance energy transfer (FRET) [30], [31] and bimolecular fluorescence complementation (BiFC) [32], [33]. In the case of BiFC, a fluorescent protein is split into two non-fluorescent fragments that are fused to a pair of interacting proteins. Interaction of the two proteins brings the split fragments into close proximity, resulting in reassembly of the fluorescent protein. Hence, reconstituted fluorescence is coupled to the interaction of the two proteins and can be used to conveniently determine how, when and where two proteins interact inside living cells. The power of this technique for capturing interactions along the secretory pathway of mammalian cells was first demonstrated by Michnick and coworkers [34]. In a similar vein, we demonstrate here that BiFC enabled visualization of a wide range of protein-protein interactions that constitute early steps in the Tat translocation cycle. We focused on interactions that had previously been established by alternative techniques or for which previous studies had led to conflicting results. These included: (i) the binding between soluble proteins such as the Tat substrate dimethyl sulfoxide reductase (DmsA) with its dedicated proofreading chaperone DmsD; (ii) the assembly of transmembrane proteins such as TatA with itself or TatB with TatC; and (iii) the targeting of soluble proteins to transmembrane subunits such as DmsA docking on TatC. Our results confirm that BiFC is a powerful tool for molecular dissection of key mechanistic steps of the Tat export process and provide the first robust screening platform of protein-protein interactions along this important pathway.

## Results

### Development of BiFC for Tat Substrate-Chaperone Interactions

**Figure 1:**
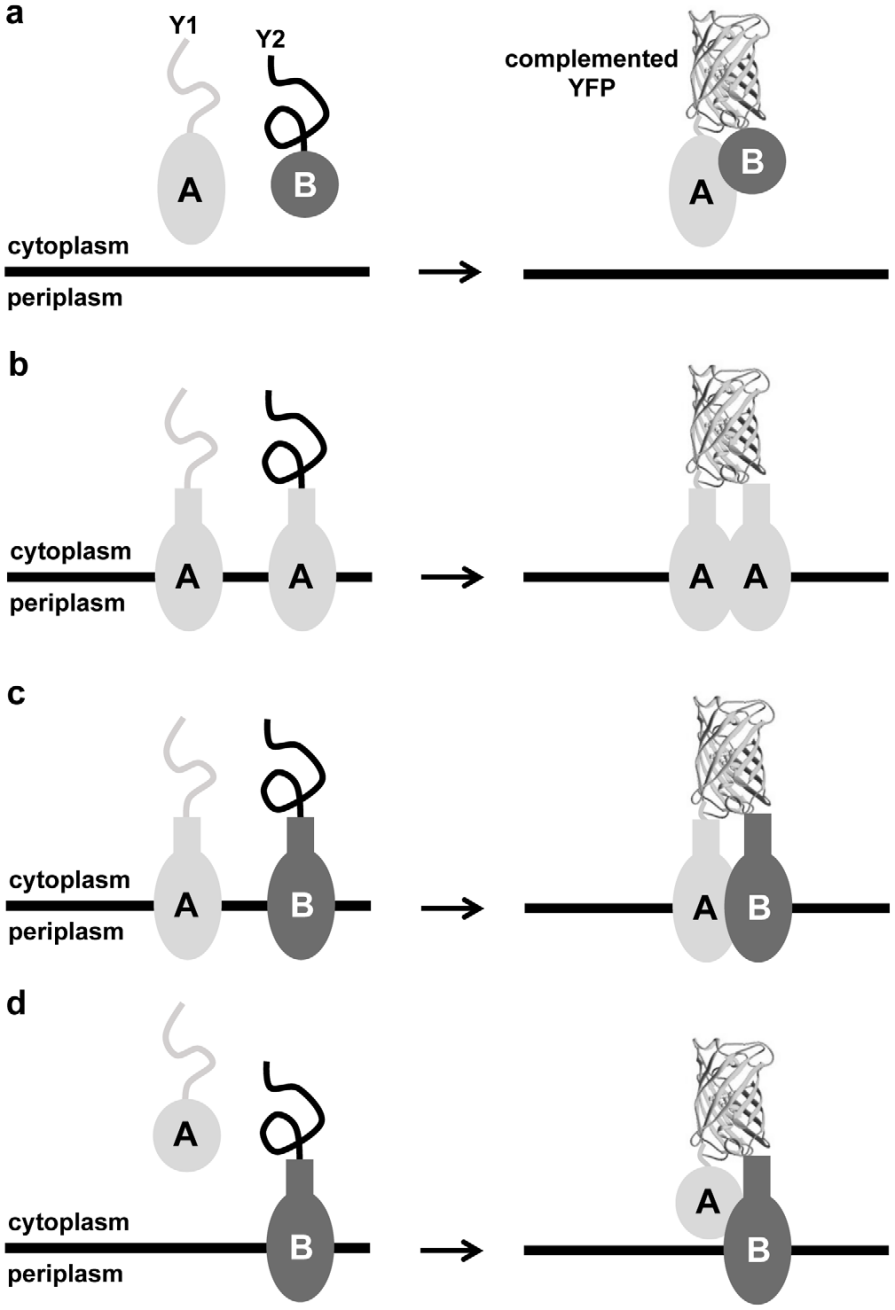
Protein interactions detected via BiFC along the Tat pathway of *E. coli*. Splitting YFP into fragments Y1 and Y2 can be used to visualize interactions between: (a) two soluble cytoplasmic proteins; (b) a transmembrane protein with itself; (c) two different transmembrane proteins; and (d) a soluble cytoplasmic protein and a transmembrane protein.

**Figure 2:**
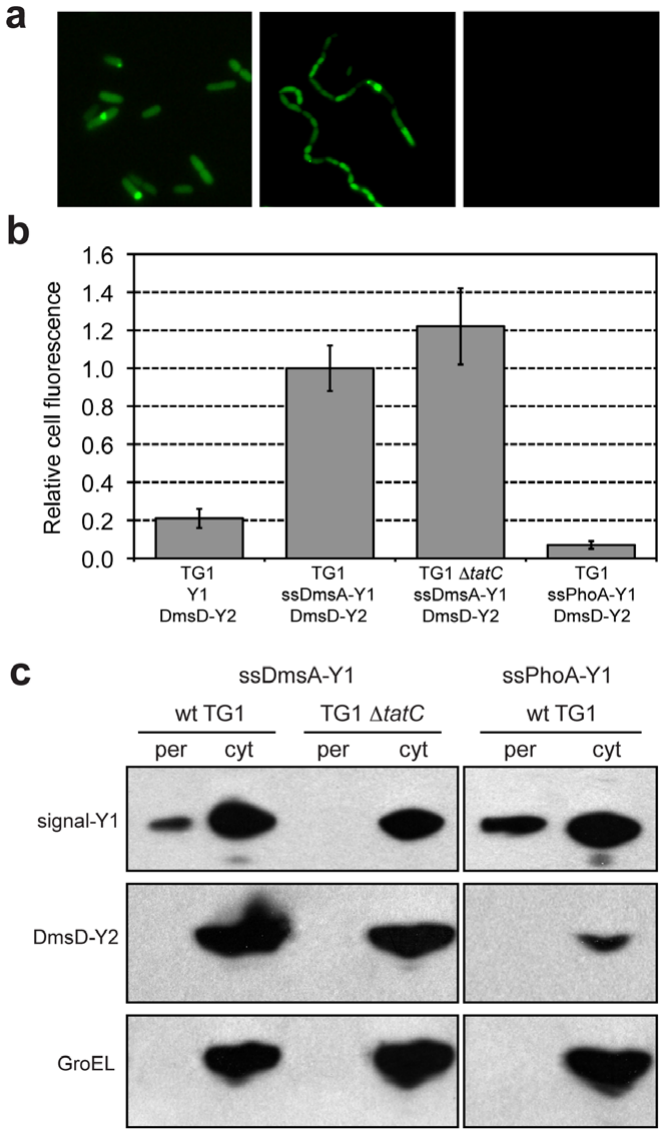
BiFC illuminates DmsA-DmsD interaction. (a) Fluorescence microscopy of wt TG1 cells expressing ssDmsA-Y1/DmsD-Y2 (left), TG1 Δ*tatC* cells expressing ssDmsA-Y1/DmsD-Y2 (center), and wt TG1 cells expressing ssPhoA-Y1/DmsD-Y2 (right). (b) Flow cytometric analysis of cells expressing constructs as indicated. Median fluorescence was obtained for each cell population and normalized to the median fluorescence measured for TG1 cells expressing ssDmsA-Y1/DmsD-Y2 (median fluorescence value for this interaction was M = 2247). Data was reported as the average of 6 replicate experiments (*n = *6) and error bars represent the standard error of the mean (sem). (c) Western blot analysis of periplasmic (per) and cytoplasmic (cyt) fractions from wt TG1 or TG1 Δ*tatC* cells expressing ssDmsA-Y1/DmsD-Y2 or ssPhoA-Y1/DmsD-Y2 as indicated. YFP1 was detected by virtue of a C-terminal FLAG tag using anti-FLAG antibody. YFP2 and GroEL proteins were detected using anti-GFP or anti-GroEL antibodies, respectively.

To visualize protein interactions between soluble and transmembrane factors that participate in various steps of the Tat export process, we employed BiFC based on split fragments of enhanced yellow fluorescent protein (YFP). Our first target was the well-characterized interaction between *E. coli* DmsA and its cognate binding chaperone DmsD (Fig. 1a). The DmsD chaperone recognizes the DmsA twin-arginine signal peptide [26] and helps orchestrate the biogenesis and assembly of the DmsA enzyme [35]. It has been suggested that this interaction serves as a proofreading step that prevents premature export of incompletely folded DmsA [36], [37]. Since the DmsA signal peptide (ssDmsA) alone is sufficient to interact with DmsD [26], we first tested whether ssDmsA fused to the N-terminal YFP fragment (ssDmsA-Y1) interacted with DmsD fused to the C-terminal YFP fragment (DmsD-Y2). As evidenced by fluorescence microscopy, wt TG1 cells expressing these two chimeras emitted strong fluorescence (Fig. 2a) that was nearly 5 times brighter than the background from control cells co-expressing an unfused version of Y1 with DmsD-Y2 (Fig. 2b). The low levels of background fluorescence observed for control cells was likely due to self-assembly of the YFP fragments in the cytoplasm. An equally strong fluorescent phenotype was observed when the same constructs were expressed in a Δ*tatC* derivative of TG1 that is incapable of Tat-specific transport (Fig. 2a and b), indicating that the interaction was not dependent on a functional Tat system (see below). Importantly, when ssDmsA was replaced with the Sec-dependent PhoA signal peptide (ssPhoA), no fluorescence above background was observed (Fig. 2a and b) verifying that the fluorescence seen following co-expression of ssDmsA-Y1 and DmsD-Y2 was highly specific for the ssDmsA-DmsD interaction. It is noteworthy that replacement of the YFP fragments with similarly designed fragments derived from a monomeric variant of RFP [38] gave nearly identical complementation results for the ssDmsA-DmsD interaction (Fig. S1). This suggests that the BiFC signal seen above was due to the specificity of this tandem chaperone/signal peptide system and was not an artifact of the split reporter protein.

To address whether the engineered ssDmsA-Y1 chimera was still faithfully recognized and exported to the periplasm by the Tat translocase, we determined the subcellular location of ssDmsA-Y1 following its co-expression with DmsD-Y2 in wt or Δ*tatC* cells. As expected, a portion of the ssDmsA-Y1 was localized to the periplasm in wt cells but not in *tatC-*deficient cells (Fig. 2c). For comparison, DmsD-Y2 was observed exclusively in the cytoplasm of both these strains (Fig. 2c). It should be noted that the Sec-dependent substrate ssPhoA-Y1, like its ssDmsA-Y1 counterpart, accumulated in both the cytoplasm and the periplasm of wt cells (Fig. 2c), indicating that the lack of YFP complementation for the ssPhoA-Y1 construct was not due to poor expression/stability or to highly efficient translocation via the Sec pathway. Taken together, these results indicate that ssDmsA-Y1 is capable of transiting the Tat pathway.

### Tat Substrate-Chaperone Interactions Do Not Require the TatABCE Proteins

**Figure 3:**
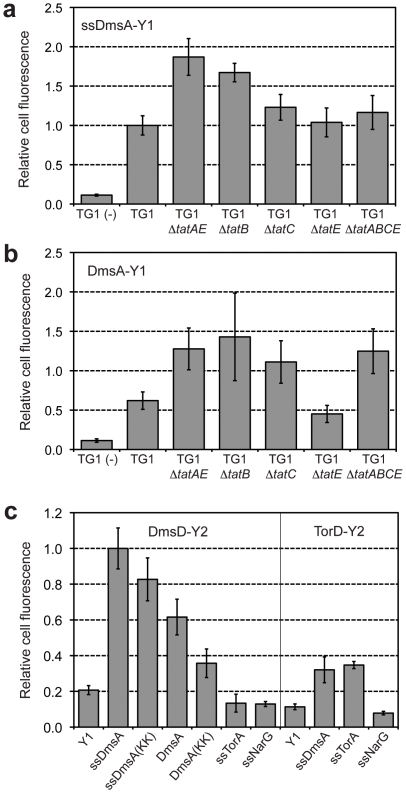
Specificity determinants of substrate/chaperone interactions. Co-expression of (a) ssDmsA-Y1/DmsD-Y2 or (b) DmsA-Y1/DmsD-Y2 in various TG1 *tat* deletion strains as indicated. TG1 (−) indicates cells that co-expressed Y1 lacking the ssDmsA signal peptide and DmsD-Y2. Median cell fluorescence was obtained via flow cytometry and normalized to that for wt TG1 cells co-expressing ssDmsA-Y1/DmsD-Y2. Data was reported as the average of 6 replicate experiments (*n = *6) and error bars represent the sem. (c) Co-expression of DmsD-Y2 with wt ssDmsA-Y1, full-length DmsA-Y1, or twin-lysine (KK) variants of ssDmsA-Y1 or DmsA-Y1 in a wt TG1 background. Chaperones DmsD and TorD each fused to Y2 were co-expressed with their cognate or non-cognate signal sequences (ssDmsA-Y1, ssTorA-Y1, ssNarG-Y1). Median cell fluorescence was obtained via flow cytometry and normalized to that for wt TG1 cells co-expressing ssDmsA-Y1/DmsD-Y2. Data was reported as the average of 6 replicate experiments (*n = *6) and error bars represent the sem.

We next sought to determine whether binding of DmsA by its cognate chaperone DmsD required the TatABCE proteins that comprise the translocase or instead was uncoupled from these components. Following co-expression of ssDmsA-Y1 and DmsD-Y2 in various *tat*-deficient strain backgrounds, we observed significant binding of ssDmsA by DmsD even when the Tat system was partially (Δ*tatE*) or completely (Δ*tatB*, Δ*tatC*, Δ*tatAE* and Δ*tatABCE*) inactivated (Fig. 3a). In addition to the ssDmsA-Y1 reporter protein, we constructed a chimera comprised of the entire DmsA enzyme (DmsA-Y1) to determine if the BiFC could be used to evaluate the binding of full-length Tat substrates by proofreading chaperones. Co-expression of DmsA-Y1 with DmsD-Y2 in wt cells resulted in a fluorescent signal that was significantly above background but only about 50% of that observed for the ssDmsA-Y1/DmsD-Y2 pair (Fig. 3b). We attribute this decrease to the lower level of soluble expression observed for the full-length DmsA-Y1 construct compared to ssDmsA-Y1 (see Fig. S2d). Similar to ssDmsA-Y1, DmsA-Y1 interacted strongly with DmsD in various *tat-*deficient mutants (Fig. 3b). Overall, our BiFC results are entirely consistent with the view that proofreading chaperones operate at an early stage of Tat export and their substrate binding activity is uncoupled from the membrane translocation step [36].

To verify that the BiFC signals from the DmsA/DmsD interactions were due to physical association between the proteins, we performed a co-purification experiment using an 8x polyhistidine-tagged version of DmsD-Y2. Co-expression of this construct with ssDmsA-Y1 or DmsA-Y1 in TG1 Δ*tatABCE* cells was performed, followed by Ni-NTA chromatography. SDS-PAGE analysis of the elution fractions collected from the column revealed that these fractions contained both the 8xHis-DmsD-Y2 and DmsA-Y1 (Fig. S2a) or ssDmsA-Y1 (data not shown). Moreover, all of the elution fractions were fluorescent, indicating that the recovered 8xHis-DmsD-Y2 was associated with ssDmsA-Y1 or DmsA-Y1 (Fig. S2b). Native PAGE analysis of the elution fractions revealed fluorescent complexes that migrated at the expected sizes for DmsA-Y1/8xHis-DmsD-Y2 and ssDmsA-Y1/8xHis-DmsD-Y2 (Fig. S2c). Western blot analysis of these same fractions confirmed the presence of the DmsA-Y1 or ssDmsA-Y2 fusion proteins in these affinity-captured complexes (Fig. S2d). Similar co-purification results were obtained using versions of DmsA or DmsD that lacked the Y1 fragments (data not shown), although the purified complexes were of course not fluorescent and the yield was lower. We suspect that the higher yield for Y1/Y2-containing complexes was the result of intermolecular stabilization or trapping afforded by the nearly irreversible assembly of the split YFP fragments. As such, the use of YFP fragments may be a convenient strategy for co-purification of interacting proteins, especially those whose association in the cell is short-lived. Overall, these results indicate that the BiFC signals observed above were due to authentic association between the substrate/chaperone pair.

### Specificity Determinants of Substrate-Chaperone Interactions

Using our BiFC system, we next explored the substrate specificity of the DmsD proofreading chaperone. First, we tested whether the twin-arginine residues in ssDmsA, which are needed for functional Tat transport, were required for DmsD binding and in turn the BiFC signal. For this, we generated variants of ssDmsA-Y1 and DmsA-Y1 in which the twin-arginine residues in the (S/T)RRxFLK consensus motif were each mutated to lysine, a substitution that completely abolishes export [39], [40]. When ssDmsA(KK)-Y1 was co-expressed with DmsD-Y2 in wt TG1 cells there was no significant difference in cell fluorescence. This result was consistent with *in vitro* binding results observed for ssTorA(KK), which displayed identical TorD binding characteristics to its twin-arginine counterpart [25]. Cells co-expressing full-length DmsA(KK)-Y1 were also fluorescent although less so than their twin-arginine counterpart (Fig. 3c), suggesting that regions of the mature portion of DmsA may play a role in DmsD specificity. Our results with both the ssDmsA and full-length DmsA constructs support the conclusion that the twin-arginine motif itself is clearly not the overarching signal recognition factor.

To determine whether the DmsD chaperone was specific for its cognate substrate or instead exhibited promiscuity as has been seen previously [26], [41], we cloned the signal peptides from the Tat substrates DmsA, TorA and NarG as fusions with the Y1 fragment. Following co-expression of these constructs with DmsD-Y2 in wt TG1 cells, a strong BiFC signal was observed only for the ssDmsA-Y1/DmsD-Y2 pair (Fig. 3c). It should be noted that the exquisite specificity observed here for DmsD was not observed in earlier studies where DmsD was reported to bind signal peptides derived from DmsA and TorA [26], [41]. However, *in vivo* complementation assays with authentic DmsA and TorA substrates revealed that DmsD and TorD cannot replace one another [42], suggesting that substrate promiscuity of DmsD may be an artifact of the experimental conditions used to investigate signal peptide-chaperone binding. Even our assay was not immune to this sort of artifact as testing of TorD-Y2 against the same set of signal peptides revealed a BiFC signal for both the cognate ssTorA-Y1 and the non-cognate ssDsmA-Y1 constructs (Fig. 3c). Nonetheless, our results provide further evidence that the BiFC strategy enables direct detection of interactions between different chaperones/signal peptide pairs directly in *E. coli* without needing to alter the geometry (e.g., linker lengths) or orientation (N- versus C-terminal) of the YFP fragments.

### Identification of Permissive Residues in DmsD Binding Pocket

To further demonstrate the utility of our BiFC assay, we attempted to isolate gain-of-function DmsD variants that bind ssDmsA more efficiently. Previous studies identified a “hot pocket” of residues in DmsD that are important for signal peptide binding [43]. In this study, a hyperbinding variant of DmsD carrying a single W87Y substitution and a lower affinity variant, DmsD(R15C/L75S), were reported. When the DmsD(R15C/L75S)-Y2 variant was co-expressed with ssDmsA-Y1, there was a clear decrease in the BiFC signal compared to wt DmsD (Fig. S3a), consistent with the earlier report. However, binding activity of DmsD(W87Y)-Y2 was indistinguishable from wt DmsD (Fig. S3a). Therefore, to experimentally identify residues in this region of DmsD that permitted signal peptide binding, we created 2 random libraries of DmsD variants using an NNK library approach that targeted residues W72/L75/F76 in the putative binding pocket [43]. The resulting DmsD libraries were screened via fluorescence-activated cell sorting (FACS) using either ssDmsA-Y1 or full-length DmsA-Y1 as the co-expressed partner. As seen in Table 1, a strong bias for hydrophobic, uncharged residues in these positions was observed, especially in positions 72 and 76 where a hydrophobic residue was found in 16/21 and 19/21 clones, respectively (7 and 10 of these, respectively, were wt in this position). Position 75 appears to be the most flexible as more than half of the clones carried a hydrophilic residue in this position, and in 2 of these cases the residue was charged (Lys, Asp). It is noteworthy that the BiFC signals emitted by all the isolated clones were comparable to the signal seen for wt DmsD(WLF)-Y2, except for DmsD(HYF) which exhibited a gain-of-function phenotype (Fig. S3a). We attribute this increased fluorescence to improved substrate binding because the expression level of each clone was unchanged relative to wt DmsD (Fig. S3c). Interestingly, much less structural variability was tolerated for these residues in DmsD when full-length DmsA-Y1 was used as substrate (Table 1). This suggests that substrate binding specificity is dependent on the context of the signal peptide and that the sequence determinants for binding of the entire native preprotein are more specific compared to the signal peptide alone. In support of this notion, the most interesting clones in the context of ssDmsA-Y1 (e.g., DmsD(R15C/L75S) and DmsD(HYF)) produced BiFC signals that were indistinguishable from wt DmsD when full-length DmsA-Y1 was the substrate (Fig. S3b).

**Table 1:** Isolation of permissive residues in the putative binding pocket of DmsD.

DmsD clone	Binding partner	Sequence	# of times isolated	ssDmsA binding activity*	DmsA binding activity*
wild-type	ssDmsA	71 AWQRLFV 77	1	1.00	0.62
HYF	ssDmsA	-H--YF-	2	1.48	0.67
YLF	ssDmsA	-Y--LF-	1	0.92	0.81
IVT	ssDmsA	-I--VT-	1	1.21	0.46
FYL	ssDmsA	-F--YL-	1	1.22	0.84
FDL	ssDmsA	-F--DL-	1	1.20	nd
FAP	ssDmsA	-F--AP-	1	0.90	nd
FQM	ssDmsA	-F--QM-	1	0.87	nd
VKM	ssDmsA	-V--KM-	1	1.09	nd
SNI	ssDmsA	-S--NI-	1	1.11	nd
SPH	ssDmsA	-S--PH-	1	1.09	nd
wild-type	DmsA	71 AWQRLFV 77	1	1.00	0.62
WMF	DmsA	-W--MF-	2	nd	0.63
WYF	DmsA	-W--YF-	2	nd	0.73
WFF	DmsA	-W--FF-	1	nd	0.59
FHL	DmsA	-F--HL-	1	nd	0.48
FHP	DmsA	-F--HP-	1	nd	0.46
FFP	DmsA	-F--FP-	1	nd	0.45

*Values are the average of six replicate experiments and the standard error of the mean (sem) is less than 15% in each case.

### Detection of Interactions between Transmembrane Components of the Tat Translocase

**Figure 4:**
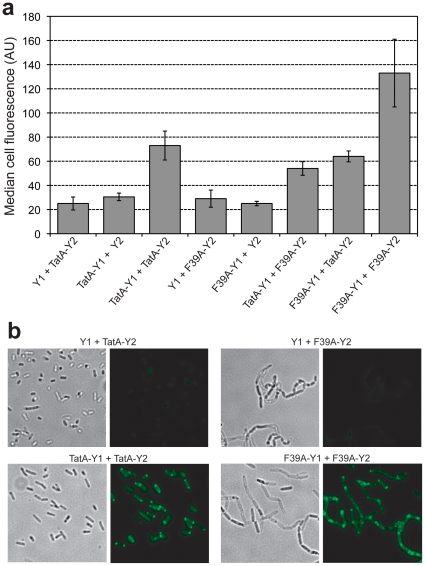
Visualizing the formation of TatA homo-oligomers. (a) Cell fluorescence of TG1 Δ*tatABCE* cells expressing TatA-Y1, TatA-Y2, F39A-Y1, F39A-Y2, and the negative controls Y1 or Y2. Median fluorescence values were obtained via flow cytometric analysis and reported as the average of 3 replicate measurements (*n = *3). Error bars represent the sem. (b) Bright field illumination and fluorescence microscopy for phenotypic analysis of chain complementation and fluorescence localization in TG1 Δ*tatAE* cells expressing various TatA chimeras as indicated.

Previous studies have established that each of the Tat components form stable, defined, homo-multimeric complexes [13], [14], [44]–[48]. Hence, we next tested whether BiFC could be used to detect interactions between the integral TatABC membrane proteins that comprise the translocase and are essential for Tat export (see Fig. 1b and c). For these experiments, each Tat gene was cloned as a fusion to both Y1 and Y2 (e.g., TatA-Y1 and TatA-Y2) and expressed in TG1 cells lacking the *tatABCE* genes. We chose a strain background lacking all *tat* genes because previous studies have shown that self-assembly of individual Tat components does not strictly require any of the other Tat components [44]–[47]. In the case of TatA, we observed a BiFC signal that was 2–3 fold above the negative controls (Fig. 4a), albeit an order of magnitude lower than that seen for the ssDmsA/DmsD interaction described above. Co-expression of a TatA mutant with a substitution in the predicted amphipathic region (F39A) that blocks translocation activity and leads to aberrant TatA oligomers [49] was still able to assemble with wt TatA (Fig. 4a). Interestingly, co-expressed F39A-Y1 and F39A-Y2 were observed to homo-oligomerize very efficiently, with a BiFC signal that was nearly twice as fluorescent as the wt TatA homo-oligomers (Fig. 4a).

**Figure 5:**
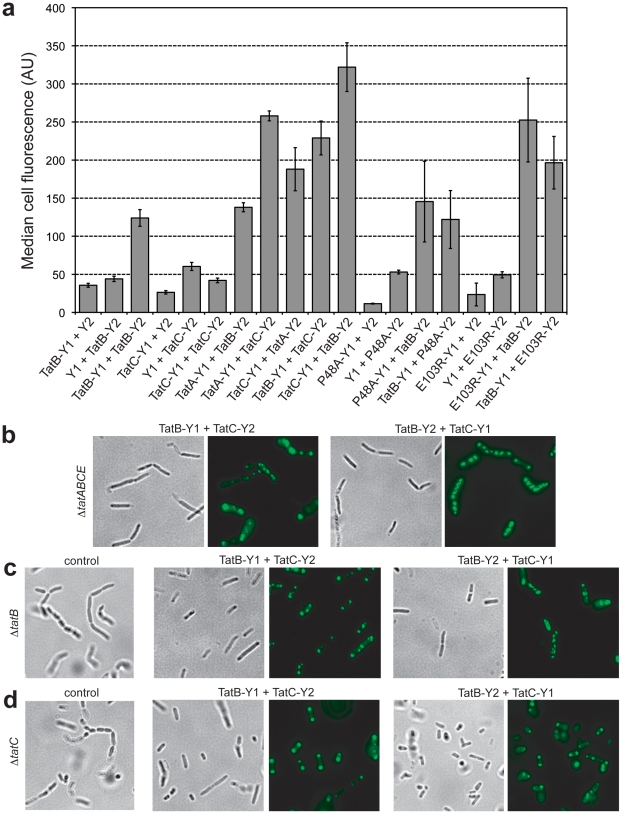
Assembly of fluorescent TatBC homo- and hetero-oligomers. (a) Cell fluorescence of TG1 Δ*tatABCE* cells expressing TatB and TatC BiFC chimeras as indicated. In addition to wt TatC, the TatC variants P48A and E103R were also evaluated. Unfused Y1 and Y2 constructs co-expressed with TatB or TatC chimeras served as negative controls. Median fluorescence values were obtained via flow cytometric analysis and reported as the average of 3 replicate measurements (*n = *3). Error bars represent the sem. Bright field illumination and fluorescence microscopy for (b) TG1 Δ*tatABCE*, (c) TG1 Δ*tatB* and (d) TG1 Δ*tatC* cells co-expressing TatB-Y1/TatC-Y2 or TatB-Y2/TatC-Y2 as indicated. Also shown are plasmid-free TG1 Δ*tatB* and Δ*tatC* cells (control) to illustrate the chain phenotype of Tat-deficient mutants.

To test assembly of the TatA BiFC constructs under more physiologically relevant conditions, we co-expressed TatA-Y1/TatA-Y2 in TG1 Δ*tatAE* cells that express native TatB and TatC from the chromosome. This resulted in a clear BiFC signal compared to controls (Fig. 4b) that was quantitatively similar to the BiFC signal seen in Δ*tatABCE* cells (data not shown). It should be noted that the fluorescence appeared predominantly at the cell poles, consistent with earlier TatA labeling studies [50]. To confirm that the TatA-Y1 and TatA-Y2 chimeras were able to form functional translocases, we examined these cells by light microscopy. It is well known that *tat-*deficient strains form chains of up to 15 cells [51]. This cell division defect results from the mislocalization of two Tat-dependent amidases, AmiA and AmiC, which have been implicated in cleavage of the septum during cell division [52]. We observed that TatA-Y1 and TatA-Y2 are able to form functional translocases with endogenous TatB and TatC as evidenced by the ability of these constructs to reverse the chain phenotype of Δ*tatAE* cells (Fig. 4b). In contrast, co-expression of TatA(F39A)-Y1 and TatA(F39)-Y2 did not reverse the chain phenotype of Δ*tatAE* cells (Fig. 4b), even though these constructs yielded strong BiFC fluorescence that accumulated at the cell poles of Δ*tatABCE* cells (Fig. 4a). Taken together, these results indicate that the export defect of TatA(F39A) mutants does not arise from an inability of to self-assemble. Similar self-assembly studies were performed for TatB and TatC. Co-expression of TatB-Y1/TatB-Y2, but not TatC-Y1/TatC-Y2, in Δ*tatABCE* cells resulted in a BiFC signal (Fig. 5a). The lack of BiFC for TatC-Y1/TatC-Y2 was not attributable to instability or inactivity of the TatC fusions, or low production of TatC caused by its overexpression [47], because these chimeras formed functional translocases as evidenced by their ability to reverse the chain phenotype of Δ*tatC* cells (data not shown).

We next investigated the formation of hetero-multimeric complexes among the various Tat components. Co-expression of different pairs of Tat components (e.g., TatA-Y1 + TatC-Y2) in cells lacking the native *tat* genes resulted in strong BiFC signals for TatA-Y1/TatC-Y2 and TatB-Y1/TatC-Y2 and a weaker signal for the TatA-Y1/TatB-Y2 that were all 3–4 times more fluorescent than the respective negative controls (Fig. 5a). These results were entirely consistent with earlier findings that TatB and TatC form a complex containing multiple copies of each subunit [12] that serves as the binding site for Tat substrates [17], [53], and that TatB is capable of interacting with TatA even in the absence of TatC [14]. Importantly, switching the Y1 and Y2 fusion partners corroborated the BiFC signals measured for TatA-Y2/TatC-Y1 and TatB-Y2/TatC-Y1 (Fig. 5a). However, the TatA-Y2/TatB-Y1 signal was indistinguishable from the control (data not shown). This result together with the relatively low signal seen above for TatA-Y1/TatB-Y2 suggests that the TatA-TatB interaction may be considerably weaker than that of TatA-TatC and TatB-TatC. We also tested two TatC variants: TatC(P48A) carries a substitution in the first periplasmic loop region that abolishes export and partially impairs TatC interaction with TatB [49] and TatC(E103R) has a substitution in the first cytoplasmic loop between predicted transmembrane helices II and III that blocks export but does not affect TatBC complex formation [54]. In line with these earlier observations, both the TatC(P48A) and TatC(E103R) constructs produced BiFC signals when co-expressed with TatB, however the TatC(P48A) signal was weaker than the signals measured for the interaction between TatB and either wt TatC or TatC(E103R) (Fig. 5a).

Fluorescence microscopy revealed that TatBC assemblies were co-localized at the cell poles in both Δ*tatABCE* mutants and in the single Δ*tatB* or Δ*tatC* deletion strains (Fig. 5b–d). This is the first evidence of a polar location for TatBC complexes and is consistent with earlier findings for the individual TatB and TatC proteins [50]. Also evident in the microscopy analysis is the fact that all TatB and TatC chimeras were able to complement the corresponding single deletion strains (Fig. 5c and d). This complementation required co-expression of both TatB and TatC at nearly equal levels as independent expression of TatB or TatC chimeras was unable to complement the chain phenotype of the Δ*tatB* or Δ*tatC* cells, respectively (data not shown). Taken together, these results indicate that Tat function was not impaired under conditions of productive BiFC, but was highly sensitive to the TatBC stoichiometry.

### DmsA and DmsD Interact with the TatB and TatC Proteins

**Figure 6:**
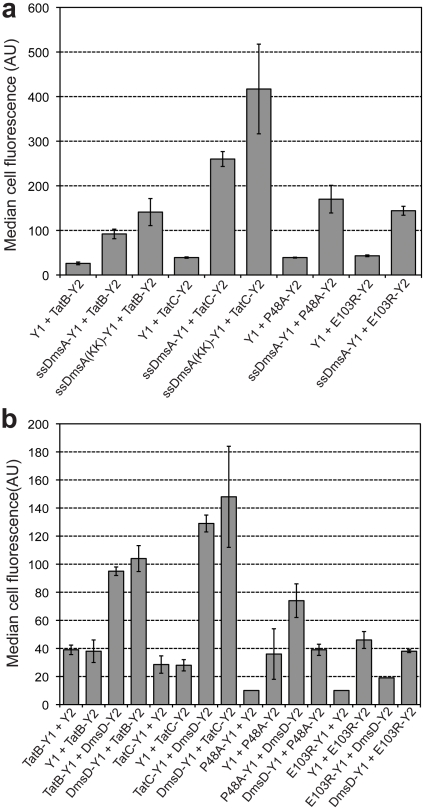
BiFC reveals substrate and chaperone “docking” on TatB or TatC. (a) Cell fluorescence of TG1 Δ*tatABCE* cells co-expressing ssDmsA-Y1 with either TatB-Y2 or TatC-Y2 as indicated. Also shown are data for the ssDmsA twin-lysine (KK) variant and the TatC variants P48A and E103R. (b) TG1 Δ*tatABCE* cells co-expressing DmsD with either TatB or TatC chimeras as indicated. Unfused Y1 and Y2 constructs co-expressed with TatB or TatC chimeras served as negative controls. Median fluorescence values were obtained via flow cytometric analysis and reported as the average of 3 replicate measurements (*n = *3). Error bars represent the sem.

As mentioned above, the TatBC complex has been implicated as the substrate-binding site [17], [53] and also as a possible docking site for the DmsD chaperone [55]. Accordingly, we next investigated interactions between the TatABC inner membrane proteins and soluble cytoplasmic factors (see Fig. 1d). Following co-expression of ssDmsA-Y1 with TatB-Y2 and TatC-Y2 in TG1 Δ*tatABCE* cells, a BiFC signal was observed that was 3.5- and nearly 7-fold above background, respectively (Fig. 6a). A much weaker but still significant BiFC signal was observed for full-length DmsA-Y1, especially when co-expressed with TatC-Y2 (data not shown). No signal above background was observed when ssDmsA-Y1 was co-expressed with TatA-Y2 (data not shown). To independently confirm the ssDmsA-Y1/TatC-Y2 interaction, we isolated membrane fractions from Δ*tatABCE* cells and analyzed these by Western blotting. When ssDmsA-Y1 was expressed alone, we detected the fusion protein in the soluble fraction but not in the membrane fraction (Fig. S4). However, when TatC-Y2 was co-expressed, the ssDmsA-Y1 construct was found to co-localize in the membrane fraction (Fig. S4) presumably due to its association with TatC-Y2.

Interestingly, when the twin arginines in ssDmsA were substituted with lysines, the BiFC signal following co-expression of TatB-Y2 and TatC-Y2 increased to 5.4- and 10.7-fold above background, respectively (Fig. 6a). This corroborates the recent observation that the twin-arginine residues of the Tat consensus motif are not essential for binding of precursor to the TatBC complex [54]. This result is also consistent with the observation above that cytoplasmic accumulation of non-exported substrates (e.g., ssDmsA(KK)) resulted in a stronger BiFC signal. When either TatC(P48)-Y2 or TatC(E103R)-Y2 was co-expressed with ssDmsA-Y1, we observed a BiFC signal that was nearly 2-fold less fluorescent than that seen for wt TatC. In the case of TatC(E103R), the reduced BiFC signal was in close agreement with recent data indicating that this variant exhibits reduced substrate binding [54]. To test whether TatBC also interacts with the DmsD chaperone, we co-expressed TatB-Y1 or TatC-Y1 with DmsD-Y2 in Δ*tatABCE* cells. DmsD interaction with TatB or TatC resulted in a ∼2-4-fold increase in the BiFC signal above background that was independent of the orientation of the split fragments (Fig. 6b). Interestingly, the P48A substitution resulted in a reduced BiFC signal while the E103R mutation blocked interaction with DmsD (Fig. 6b), suggesting that these residues, especially E103R, are important for the interaction of DmsD with the TatC subunit of the translocase.

## Discussion

Bacterial protein export requires a wide range of protein interactions between soluble and transmembrane proteins, many of which have been difficult to detect using traditional approaches especially in the context of living cells. Here, we show that the YFP BiFC overcomes these limitations and enables a detailed analysis of numerous protein-protein interactions along the bacterial Tat pathway of live cells. Among these were interactions between (i) soluble cytoplasmic proteins, (ii) transmembrane proteins with themselves or a different transmembrane protein; and (iii) soluble cytoplasmic proteins and transmembrane proteins. Although not tested here, we anticipate that specific interactions between two soluble periplasmic proteins or a periplasmic protein with a transmembrane protein will also be detectable by protein fragmentation analysis. The challenge with detecting interactions on the periplasmic side of the inner membrane is that YFP and its relatives (e.g., GFP, CFP) do not attain a fluorescent conformation in the periplasm [56] unless delivered there in an already folded conformation via the Tat system [40], [57], [58]. Hence, assembly of split fluorescent proteins in the periplasm may not yield a fluorescent signal. One solution is to use split mRFP [38] (and Fig. S1) since full-length mRFP can fold into a fluorescent conformation in the *E. coli* periplasm (our unpublished observations). Alternatively, one could employ other protein fragment complementation systems such as split β-lactamase that are compatible with assembly and folding in the periplasm [59]. It is also noteworthy that, even though not a problem in our studies, the BiFC system could be further improved by increasing the solubility of the split YFP fragments, especially Y1, using protein engineering strategies. The reduced solubility of Y1 fusion proteins can be partially offset by expressing these from a high-copy vector while co-expressing Y2 chimeras from a low-copy vector, a strategy that was used here and elsewhere [60].

We have shown that the BiFC system can be an effective tool for confirming hypotheses regarding the Tat mechanism as well as for generating new experimental insights on how the Tat system functions. For instance, it is now generally accepted that several layers of quality control regulate the export of Tat substrate proteins [61]. The first layer, which we were able to visualize, is the association of specific molecular chaperones (e.g., DmsD, TorD) with Tat substrates. These interactions are thought to be important in substrate folding as well as in preventing premature export of improperly or incompletely folded proteins [26]–[28], [62]–[64]. Our results with ssDmsA-Y1 support the notion that substrate specificity of Tat chaperones is governed by the signal peptide, however our data also indicate that the mature domain of DmsA makes an important contribution to chaperone binding. In fact, there were a few cases where we observed measurable differences for interactions involving ssDmsA versus full-length DmsA, highlighting that care should be taken when interpreting data from chaperone binding experiments where signal peptides are used as surrogates for the full-length preprotein substrate. The involvement of chaperones has also led to the interesting hypothesis that these proteins guide their substrates to the translocase. In support of this hypothesis, biochemical studies revealed that DmsD interacted tightly with the *E. coli* inner membrane and that the TatB and TatC subunits were important for this interaction [55]. Our BiFC results confirm that DmsD interacts specifically with TatB and TatC (Fig. 6b), but not TatA (our unpublished observations). We also observed that a small fraction of the ssDmsA/DmsD complexes co-localized to the cell poles (Fig. 2a and also our unpublished observations), which is also where the TatBC receptor was observed to co-localize (Fig. 5b–d). These findings provide the first genetic evidence that DmsD may play a role as a targeting factor that delivers substrates to the TatBC receptor complex. To confirm this, we are currently developing a three-way BiFC-based FRET interaction system [65] to investigate whether DmsA/DmsD/TatB (or TatC) form a ternary complex in living cells. A final step prior to substrate export appears to be evaluation of a substrate's folding state by the Tat apparatus. Indeed, mounting evidence indicates that the Tat system generally discriminates against unfolded substrates [7], [10], [23], [24] (although at least two exceptions exist [16], [66]) and it has been suggested that this folding quality control may be performed directly by the Tat translocase [24], [29]. Thus, although not directly investigated here, we anticipate that our BiFC system will enable genetic dissection of this poorly understood aspect of Tat protein export and should provide some insights into the path of a Tat precursor following its recognition by TatBC up to a step where it is brought into close vicinity of TatA.

## Materials and Methods

### Bacterial Strains, Plasmids, Growth and Induction Conditions

The bacterial strains and plasmids used in this study are described in Table 2. For cloning purposes, *E. coli* MC4100 cells were grown aerobically in either liquid LB media or on solid LB media with agar (LBA). For the BiFC assay, TG1 cells were made electrocompetent by standard methods [67], transformed with equal plasmid concentrations, and grown overnight on solid LB media and antibiotics (BD Diagnostic Systems) at 37°C. The next morning individual colonies were picked from the plates, placed into 3 mL of liquid LB with antibiotics in 16–18 mm culture tubes, and grown aerobically for 4 hrs at 37°C and 200 rpm until the optical density reached OD_600_ ∼0.5. Isopropyl β-D-thiogalactoside (IPTG) was added to a final concentration of 1 mM for induction of protein expression, the culture was then moved to a room temperature incubator (20–24°C) at 200 rpm for the next 8 hrs. Fluorescence was only measured for cells grown at room temperature. All single knockout TG1 Tat mutants were generated by P1 transduction from the Keio collection [68]. Strain TG1 Δ*tatABCE* was first created by P1 transduction of Δ*tatE*::Kan^R^ from the Keio collection; the kanamycin resistance was removed as described previously [69], and P1 transduction was performed again from BW25113 Δ*tatABC::aac* [70], however the apramycin resistance was not removed. Antibiotic selection was maintained for all markers on plasmids at the following concentrations: ampicillin (Amp), 100 µg/mL; chloramphenicol (Cam), 20 µg/mL; kanamycin (Kan), 50 µg/mL; and tetracycline (Tet), 10 µg/mL.

**Table 2:** Strains and plasmids used in this study.

Strain or Plasmid	Description	Reference
**Strain**		
MC4100	F' *araD139* Δ(*argF-lac*)*U169 rpsL150* (Str^r^) *relA1 flbB5301 deoC1 ptsF25 rbsR*	Laboratory stock
TG1	F' *traD36 lacI^q^*Δ(*lacZ*) *M15 proA^+^B^+^/supE* Δ(*hsdM-mcrB*)*5* (*rk- mk- McrB^−^*) *thi* Δ(*lac-proAB*)	Laboratory stock
TG1 Δ*tatAE*	TG1 derivative lacking the *tatA* and *tatE* genes	This study
TG1 Δ*tatB*	TG1 derivative lacking the *tatB* gene	This study
TG1 Δ*tatC*	TG1 derivative lacking the *tatC* gene	This study
TG1 Δ*tatE*	TG1 derivative lacking the *tatE* gene	This study
TG1 Δ*tatABCE*	TG1 Δ*tatE* derivative with an apramycin marked deletion Δ*tatABC::aac*	This study
**Plasmid**		
pUT18	Plasmid containing T18 fragment of the catalytic domain of *B. pertussis* adenylate cyclase; Amp^R^	^69^
pKNT25	Plasmid containing T25 fragment of the catalytic domain of *B. pertussis* adenylate cyclase; Kan^R^	^69^
pDmsALT18	*E. coli dmsA* signal peptide inserted into pUT18	^46^
pDmsDT25	*E. coli dmsD* coding sequence inserted into pKNT25	^46^
pssDmsA-Y1	pDmsALT18 with T18 sequence replaced by sequence encoding YFP N-terminal fragment; FLAG tag epitope at 3′ end	This work
pDmsD-Y2	pDmsDT25 with T25 sequence replaced by sequence encoding YFP C-terminal fragment	This work
p8xHis-DmsD-Y2	pDmsDT25 with N-terminal 8x polyhistidine tag and T25 sequence replaced by sequence encoding YFP C-terminal fragment	This work
pY1	Control plasmid expressing Y1-FLAG; made by removing *dmsA* signal peptide sequence from pssDmsA-Y1	This work
pY2	Control plasmid expressing Y2; made by removing DmsD from pDmsD-Y2	This work
pssPhoA-Y1	pDmsA-Y1-FLAG with *dmsA* signal peptide sequence replaced by DNA encoding the signal peptide of the *E. coli phoA* gene	This work
pDmsA-Y1	pssDmsA-Y1 with *dmsA* signal peptide sequence replaced by full-length *E. coli dmsA*; FLAG tag epitope at 3′ end	This work
pssDmsA(KK)-Y1	pssDmsA-Y1 with RR to KK substitution	This work
pDmsA(KK)-Y1	pDmsA-Y1 with RR to KK substitution	This work
pDnaK-Y2	pDmsD-Y2 with *dmsD* replaced by *E. coli dnaK* sequence	This work
pTorD-Y2	pDmsD-Y2 with *dmsD* replaced by the *E. coli torD* sequence	This work
pssTorA-Y1	pssDmsA-Y1 with *dmsA* signal peptide sequence replaced by *E. coli torA* signal peptide sequence	This work
pssNarG-Y1	pssDmsA-Y1 with *dmsA* signal peptide sequence replaced by *E. coli narG* signal peptide sequence	This work
pTatA-Y1	pssDmsA-Y1 with full-length *E. coli tatA* sequence in place of *dmsA* signal peptide sequence	This work
pTatA-Y2	pDmsD-Y2 with the full length *E. coli tatA* sequence in place of *dmsD*	This work
pF39A-Y1	pTatA-Y1 with F39A substitution	This work
pF39A-Y2	pTatA-Y2 with F39A substitution	This work
pTatB-Y1	pssDmsA-Y1 with full-length *E. coli tatB* sequence in place of *dmsA* signal peptide sequence	This work
pTatB-Y2	pDmsD-Y2 with the full length *E. coli tatB* sequence in place of *dmsD*	This work
pTatC-Y1	pssDmsA-Y1 with full-length *E. coli tatC* sequence in place of *dmsA* signal peptide sequence	This work
pTatC-Y2	pDmsD-Y2 with the full length *E. coli tatC* sequence in place of *dmsD*	This work
pP48A-Y1	pTatC-Y1 with P48A substitution	This work
pP48A-Y2	pTatC-Y2 with P48A substitution	This work
pE103R-Y1	pTatC-Y1 with E103R substitution	This work
pE103R-Y2	pTatC-Y2 with E103R substitution	This work
pDmsD-Y1	pssDmsA-Y1 with *dmsD* in place of *dmsA* signal peptide sequence	This work
pR1	pY1 with mRFP1 Q66T N-terminus in place of Y1; FLAG epitope at 3′ end	This work
pR2	pY2 with mRFP1 Q66T C-terminus in place of Y2	This work
pssDmsA-R1	pssDmsA-Y1 with N-terminus of mRFP1 Q66T in place of *dmsA* signal peptide sequence	This work
pDmsD-R2	pDmsD-Y2 with C-terminus of mRFP1 Q66T in place of *dmsA* signal peptide sequence	This work
pDmsD-Y2 Tet^R^	Kan^R^ marker in pDmsD-Y2 replaced with Tet^R^ marker	This work
pY2 Tet^R^	Kan^R^ marker in pY2 replaced with Tet^R^ marker	This work
pTatB-Y2 Tet^R^	Kan^R^ marker in pTatB-Y2 replaced with Tet^R^ marker	This work
pTatC-Y2 Tet^R^	Kan^R^ marker in pTatC-Y2 replaced with Tet^R^ marker	This work
pP48A-Y2 Tet^R^	Kan^R^ marker in pP48A-Y2 replaced with Tet^R^ marker	This work
pE103R-Y2 Tet^R^	Kan^R^ marker in pE103R-Y2 replaced with Tet^R^ marker	This work

### Construction of Plasmids

Plasmid pDmsDT25 was constructed previously by amplifying *E. coli dmsD* from genomic DNA via PCR and cloning into the *Xba*I and *Kpn*I sites of pKNT25 [43]. The resulting plasmid harbors a chimeric gene encoding *dmsD* fused to the T25 fragment of the catalytic domain of *Bordetella pertussis* adenylate cyclase. Similarly, plasmid pDmsALT18 was constructed previously by cloning a PCR fragment encoding the signal peptide of *E. coli dmsA* (excluding the signal peptide cleavage site) into the *Pst*I and *Kpn*I sites of pUT18 [43]. To establish the BiFC assay system, PCR fragments encoding the N- (1–154 aa) and C-terminal (155–238 aa) halves of the enhanced yellow fluorescent protein (YFP), abbreviated as Y1 and Y2 respectively, were amplified from pIAF817YFP (a gift from Dr. Rolf Morosoli). Plasmids pDmsD-Y2 and pssDmsA-Y1 were constructed by replacing the T25 and T18 fragments in plasmids pDmsDT25 and pDmsALT18 with Y2 and Y1, respectively. The linker sequences used for the fusion proteins were designed based on those used by Hu *et al*. [32]. All further plasmid constructions used in this study were based on these two initial plasmids. All plasmid DNA constructs were verified by sequencing.

### Fluorescence Analysis

After induction of protein expression, flow cytometric data was collected on a FACSCalibur System (Becton Dickinson) at 0 and 8 hrs post induction. Samples for flow cytometry readings were prepared by diluting 50 µL of live bacterial cells directly from culture in 1 mL of 1x PBS. Median fluorescence was determined from histograms of the cell fluorescence emitted by 30,000 viable cells collected using the FACSCalibur flow cytometer in scan mode. For microscopy, 15 µL of live bacterial cells directly from culture were placed onto a microscope slide with cover slip. All images were taken under oil immersion microscopy using a Zeiss 100x/1,30 lens. Microscopy was performed on a Zeiss Axioskop 40 equipped with a Zeiss 100x/1,30 Oil Plan-NEOFLUAR lens, an X-Cite light source (EXFO, Mississauga, Ontario), a Semrock Brightline filter cube for YFP emission (YFP-2427A-ZHE) (Rochester, NY), digitally imaged with a SPOT FLEX digital camera (Diagnostic Instruments, Inc.) and controlled with Spot Imaging Software. All images captured under 100x-oil immersion microscopy using the Zeiss 100x/1,30 Oil Plan-NEOFLUAR lens were under bright field illumination (exposure 150 ms) or under UV illumination (exposure 500 ms). For RFP analysis, see Supplemental Methods S1.

#### DmsD NNK library construction and testing

Random DmsD libraries were constructed by introducing diversity to the W72/L75/F76 residues of the DmsD protein. Briefly, site-directed random mutagenesis (Stratagene QuickChange® Site-Directed Mutagenesis Kit) of these residues was performed using degenerate NNK primers to amplify *dmsD* from plasmid pDmsD-Y2. The resulting DNA library was transformed into XL-1 Blue cells and ∼10^5^ clones (>3x coverage) were obtained. Library cells were harvested, grown in liquid culture and plasmid DNA was isolated. The isolated plasmid DNA library was digested with *Sph*I and *Kpn*I to excise the diversified *dmsD* genes, which were subsequently ligated into pDmsD-Y2 that had been similarly digested with *Sph*I and *Kpn*I to remove wild-type (wt) *dmsD*. This was done to avoid any potential mutations in the plasmid backbone that may have been introduced during the site-directed mutagenesis reaction. This library was isolated from cells and electroporated into TG1 cells that contained either pssDmsA-Y1 or pDmsA-Y1. This library was spread on LBA plates supplemented with Amp and Kan and incubated overnight at 37°C for resolution of transformants. The cells were pooled into a 500 mL culture, allowed to grow to OD_600_ ∼0.5 at 37°C and 200 rpm, induced with 1 mM IPTG at room temperature and 200 rpm for 8 hrs. Aliquots of the culture were taken and resuspended in 1 mL of 1x PBS and run through a FACSCalibur flow cytometer set for cell recovery mode. The gate used on the FACSCalibur was set to recover cells with a fluorescent signal greater than the ssDmsA-Y1/DmsD-Y2 or DmsA-Y1/DmsD-Y2 BiFC signal. The recovered cells were concentrated on 0.45 µm sterile membrane filters (Whatman) and the membrane filters were transferred to LBA + Amp/Kan plates to allow single colonies to grow overnight at 37°C. Isolates from the overnight incubation were then picked and grown in 96-well plates to an OD_600_ of ∼0.5 at 37°C and 200 rpm, induced with 1 mM IPTG at room temperature and 200 rpm for 8 hrs and then checked for fluorescence using a fluorescence microplate reader (Biotek Synergy HT) with excitation filter 485/20 and emission filter 528/20. Cells with a fluorescent signal greater than the ssDmsA-Y1/DmsD-Y2 or DmsA-Y1/DmsD-Y2 signal were grown and plasmid was harvested for DNA sequencing. Selected sequences are listed in Table 1.

#### Cell fractionation and protein analysis

After 8 hrs of induction, 1 mL of cells was collected and the OD_600_ was measured using a spectrophotometer (Thermo Scientific Biomate3). The cells were spun down for 2 min at 13,000×g and the supernatant was removed. The periplasmic fraction from the *E. coli* cells was isolated using a modified protocol of the Epicentre Biotechnologies PeriPreps™ Periplasting Kit (Madison, WI), where the periplasting buffer did not contain any Ready-Lyse Lysozyme. The soluble protein fraction from the periplasted *E. coli* cells was isolated with BugBuster® Master Mix (Novagen) according to the manufacturer's protocol. For Western blotting, an equal volume of 2x SDS-PAGE buffer was added to the periplasmic and soluble protein fractions and then boiled for 15 min at 100°C. Samples were loaded onto 4–20% iGels (NuSep Ltd, Australia) where protein amount was normalized to the optical density of the cells taken before fractionation. After SDS-PAGE, the proteins were transferred to Immobilon-P PVDF 0.45 µm membrane (Millipore, MA) and probed for the epitope FLAG (DYKDDDK) tag on all Y1 constructs using the primary antibody anti-FLAG® M2 (Stratagene, CA). To detect the Y2 fragment, the primary antibody was anti-GFP (Roche, IN). As a cytoplasmic fractionation marker, the primary antibody anti-GroEL (Sigma) was used. The secondary antibody was always anti-mouse IgG-HRP (Promega, WI). HRP detection was via chemiluminescence using the Immun-Star HRP Chemiluminescent Kit (BioRad) and captured on X-Omat Film (Kodak). For substrate/chaperone co-purification and membrane co-localization protocols, see Supplemental Methods S1.

## Supporting Information

Figure S1 RFP BiFC reports DmsA-DmsD interaction. (a) BiFC analysis using split mRFP1Q66T in wt TG1 cells. Shown are fluorescence microscopy images of TG1 cells co-expressing ssDmsA-R1 and DmsD-R2, as well as controls co-expressing unfused R1 and/or R2 as indicated. (b) Quantification of mRFP1Q66T BiFC signals using a fluorescence microplate reader for the same cells as in (a). Whole cell fluorescence values were normalized to the fluorescence emission from wt TG1 cells expressing ssDmsA-R1/DmsD-R2 and reported as the average of 3 replicate measurements (n = 3). Error bars represent the sem.
(1.62 MB TIF)

Figure S2 Co-purification of substrate/chaperone pairs from the cytoplasm of *E. coli*. (a) Purification of 8xHis-DmsD-Y2 from TG1 Δ*tatABCE* cells co-expressing DmsA-Y1. Lanes were loaded with (from left to right): MW, molecular weight ladder; 1, cell lysate; 2, 50k MWCO filtrate; 3, flow-through; 4, 5 mM imidazole; 5, 60 mM imidazole; 6, 80 mM imidazole; 7, 100 mM imidazole; 8, 150 mM imidazole; 9, 1000 mM imidazole. Numbers to the left correspond to the MW of the ladder proteins. Gel was stained with BioRad BioSafe Coomassie Blue and imaged on a BioRad ChemiDoc. (b) UV illumination of elution fractions corresponding to lanes 5–9 in (a). (c) Native PAGE analysis of elution fractions corresponding to lanes 6–9 in (a) from DmsA-Y1 expressing cells. Also shown are similar elution fractions generated from cells co-expressing ssDmsA-Y1 with 8xHis-DmsD-Y2. PAGE gel was illuminated using UV transilluminator. (d) Western blot analysis of samples in (c) using anti-FLAG antibodies that recognize the C-terminal FLAG tag on DmsA-Y1 and ssDmsA-Y1.
(3.37 MB TIF)

Figure S3 Isolation of gain-of-function chaperones. (a) Cell fluorescence of DmsD-Y2 library isolates (HYF, YLF, FYL, IVT) following co-expression with ssDmsA-Y1 in TG1 cells. Two previously characterized mutants (R15C/L75S and W87Y) were included for comparison. Unfused Y2 co-expressed with ssDmsA-Y1 served as a negative control. (b) Cell fluorescence of the same library isolates described in (a) but co-expressed with full-length DmsA-Y1 in TG1 cells. All median fluorescence values obtained via flow cytometric analysis were normalized to the signal obtained for ssDmsA-Y1/DmsD-Y2 signal. These normalized values are reported as the average of 3 replicate measurements (n = 3). Error bars represent the sem. (c) Western blot analysis of the cytoplasmic (c) or periplasmic (p) fractions isolated from cells co-expressing ssDmsA-Y1 with the DmsD-Y2 variants as indicated. GroEL served as a fractionation marker for cytoplasmic protein.
(1.00 MB TIF)

Figure S4 Co-localization of ssDmsA-Y1 with TatC in E. coli membranes. Western blot analysis of soluble and membrane fractions isolated from TG1 Δ*tatABCE* cells expressing ssDmsA-Y1 alone or co-expressing ssDmsA-Y1 with TatC-Y2. Blot was probed with anti-FLAG antibodies for detection of ssDmsA-Y1. Numbers to the left indicate the molecular weight (MW) of the ladder proteins. Two separate aliquots from the fraction collected from the top of the 70% sucrose layer (total membrane fraction) were analyzed side-by-side on the blot. An equivalent amount of soluble or membrane proteins was added to each lane.
(0.58 MB TIF)

Materials and Methods S1 Text file.
(0.05 MB DOC)
