# Supplementary figures and images for: Visualizing Interactions along the Escherichia coli Twin-Arginine Translocation Pathway Using Protein Fragment Complementation
Source: PLoS One. 2010 Feb 16;5(2):e9225. doi: 10.1371/journal.pone.0009225 (PMC2821923; doi:10.1371/journal.pone.0009225)

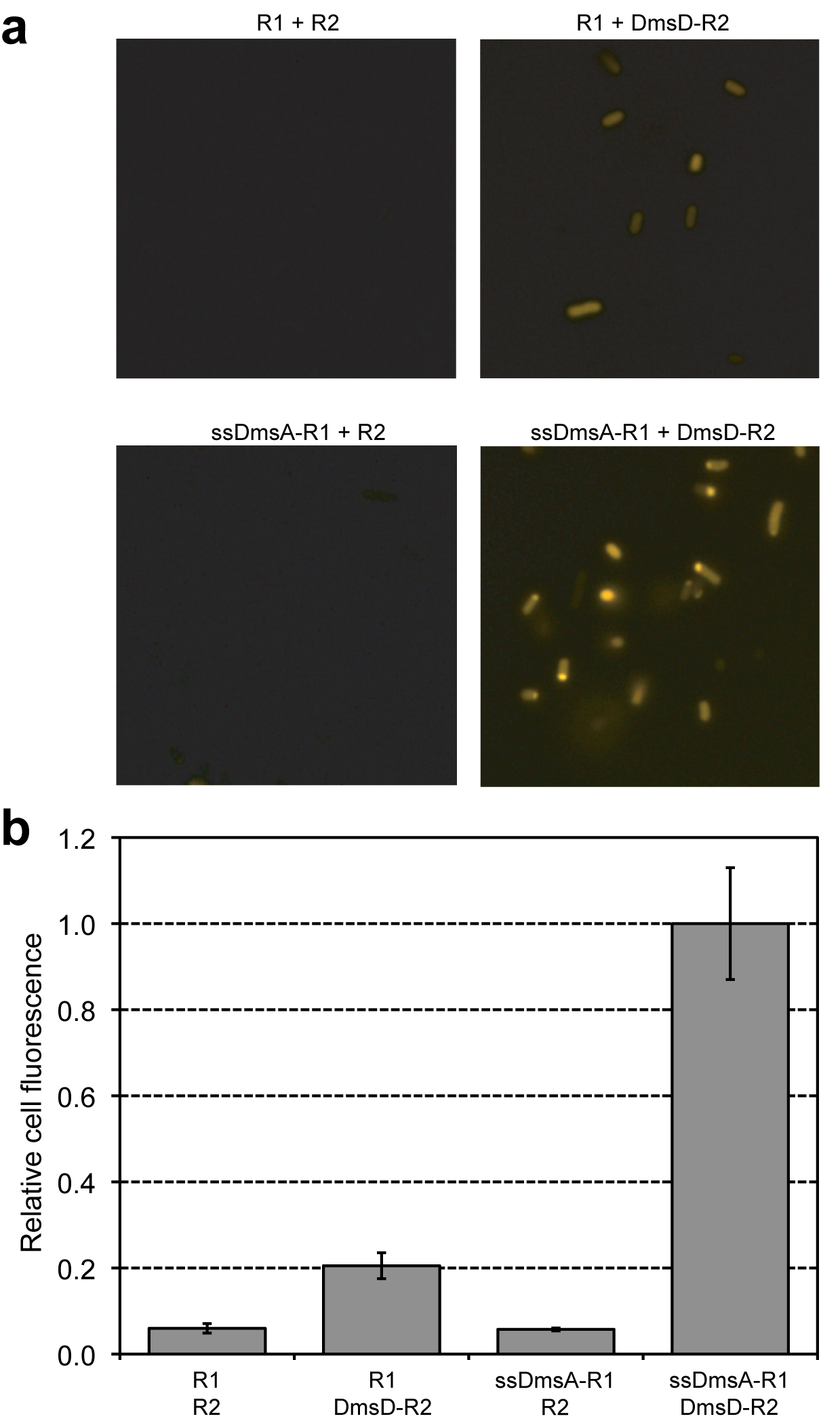

Supplement: Figure S1 — RFP BiFC reports DmsA-DmsD interaction. (a) BiFC analysis using split mRFP1Q66T in wt TG1 cells. Shown are fluorescence microscopy images of TG1 cells co-expressing ssDmsA-R1 and DmsD-R2, as well as controls co-expressing unfused R1 and/or R2 as indicated. (b) Quantification of mRFP1Q66T BiFC signals using a fluorescence microplate reader for the same cells as in (a). Whole cell fluorescence values were normalized to the fluorescence emission from wt TG1 cells expressing ssDmsA-R1/DmsD-R2 and reported as the average of 3 replicate measurements (n = 3). Error bars represent the sem. (1.62 MB TIF) [file pone.0009225.s001.tif]

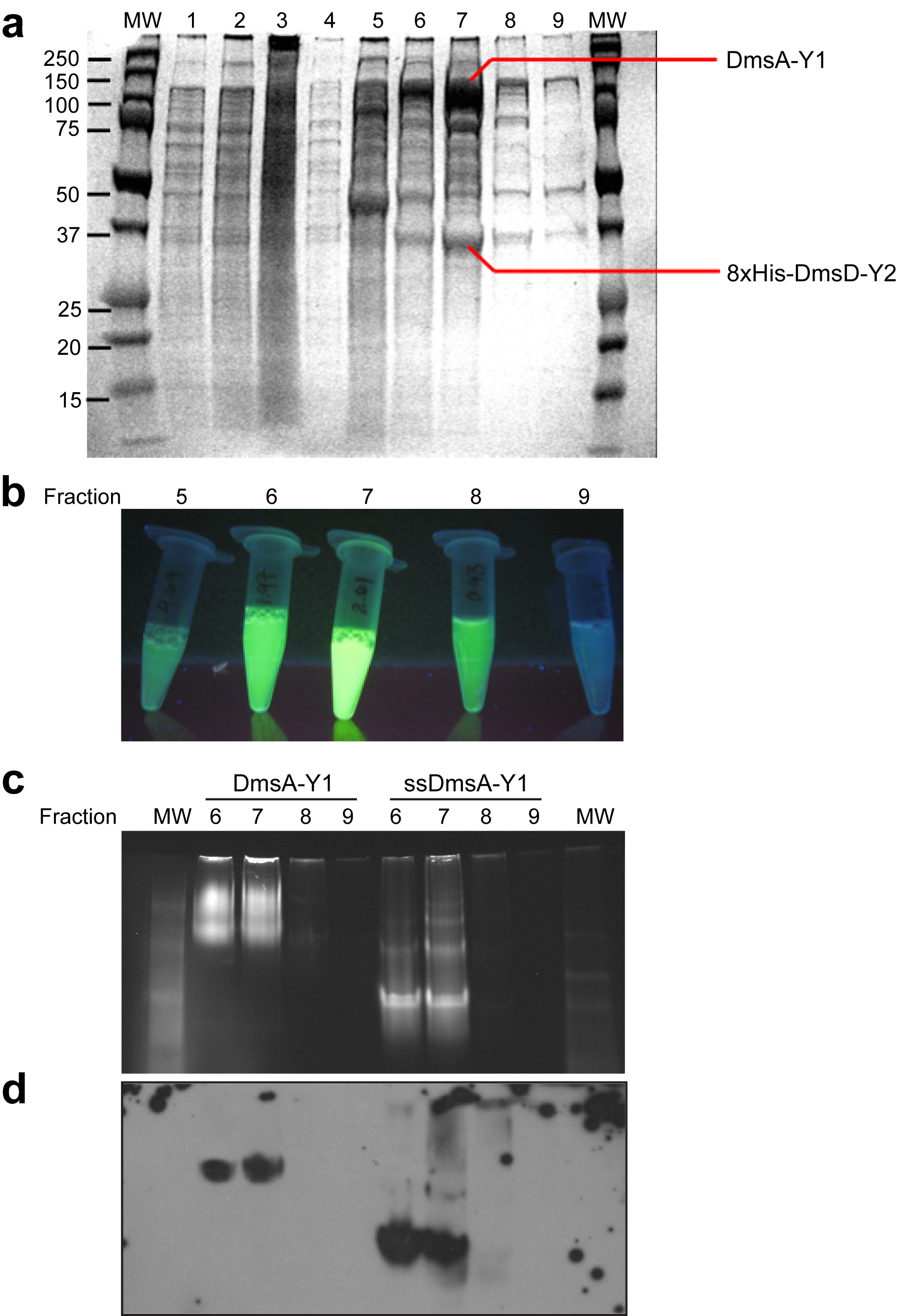

Supplement: Figure S2 — Co-purification of substrate/chaperone pairs from the cytoplasm of E. coli. (a) Purification of 8xHis-DmsD-Y2 from TG1 ΔtatABCE cells co-expressing DmsA-Y1. Lanes were loaded with (from left to right): MW, molecular weight ladder; 1, cell lysate; 2, 50k MWCO filtrate; 3, flow-through; 4, 5 mM imidazole; 5, 60 mM imidazole; 6, 80 mM imidazole; 7, 100 mM imidazole; 8, 150 mM imidazole; 9, 1000 mM imidazole. Numbers to the left correspond to the MW of the ladder proteins. Gel was stained with BioRad BioSafe Coomassie Blue and imaged on a BioRad ChemiDoc. (b) UV illumination of elution fractions corresponding to lanes 5–9 in (a). (c) Native PAGE analysis of elution fractions corresponding to lanes 6–9 in (a) from DmsA-Y1 expressing cells. Also shown are similar elution fractions generated from cells co-expressing ssDmsA-Y1 with 8xHis-DmsD-Y2. PAGE gel was illuminated using UV transilluminator. (d) Western blot analysis of samples in (c) using anti-FLAG antibodies that recognize the C-terminal FLAG tag on DmsA-Y1 and ssDmsA-Y1. (3.37 MB TIF) [file pone.0009225.s002.tif]

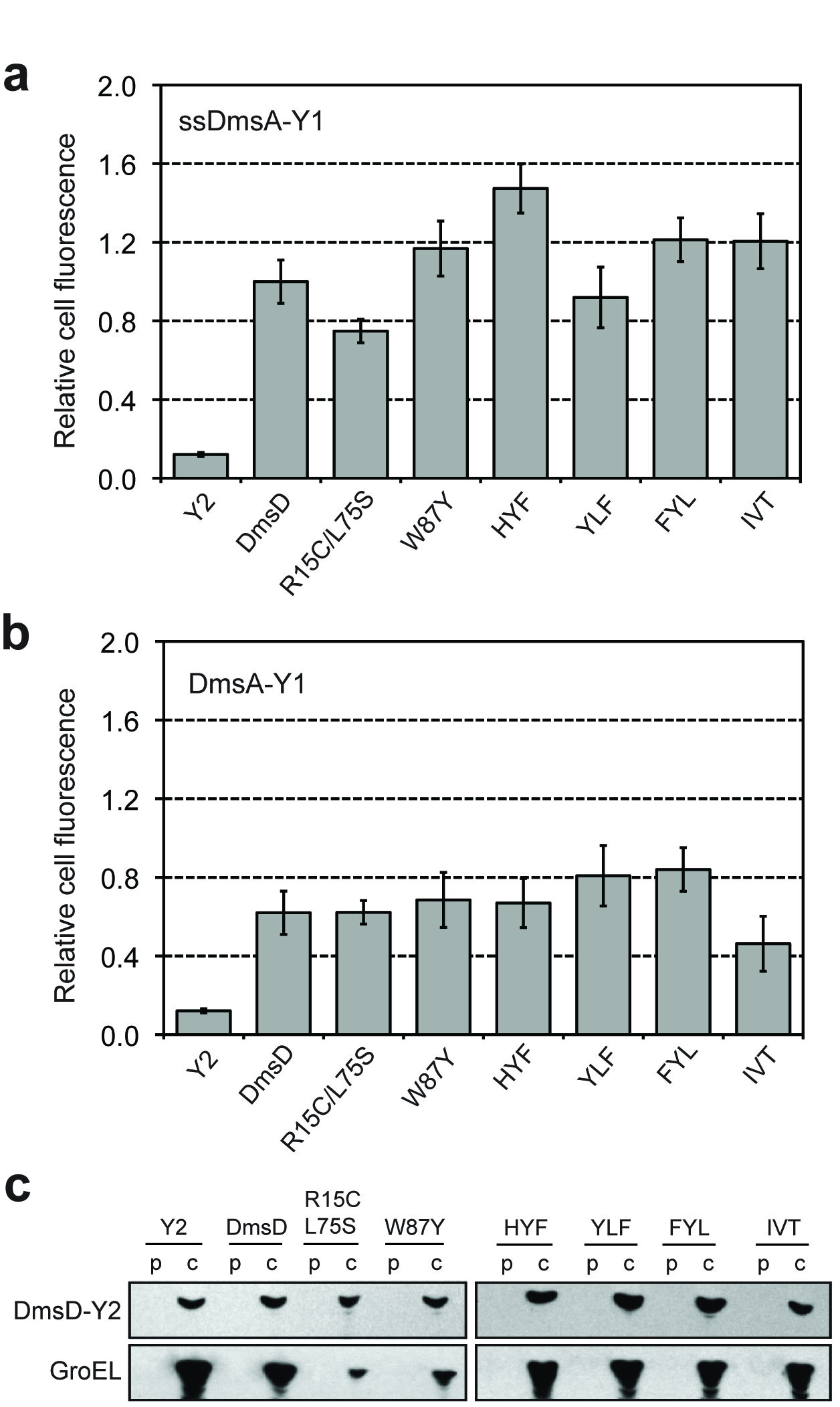

Supplement: Figure S3 — Isolation of gain-of-function chaperones. (a) Cell fluorescence of DmsD-Y2 library isolates (HYF, YLF, FYL, IVT) following co-expression with ssDmsA-Y1 in TG1 cells. Two previously characterized mutants (R15C/L75S and W87Y) were included for comparison. Unfused Y2 co-expressed with ssDmsA-Y1 served as a negative control. (b) Cell fluorescence of the same library isolates described in (a) but co-expressed with full-length DmsA-Y1 in TG1 cells. All median fluorescence values obtained via flow cytometric analysis were normalized to the signal obtained for ssDmsA-Y1/DmsD-Y2 signal. These normalized values are reported as the average of 3 replicate measurements (n = 3). Error bars represent the sem. (c) Western blot analysis of the cytoplasmic (c) or periplasmic (p) fractions isolated from cells co-expressing ssDmsA-Y1 with the DmsD-Y2 variants as indicated. GroEL served as a fractionation marker for cytoplasmic protein. (1.00 MB TIF) [file pone.0009225.s003.tif]

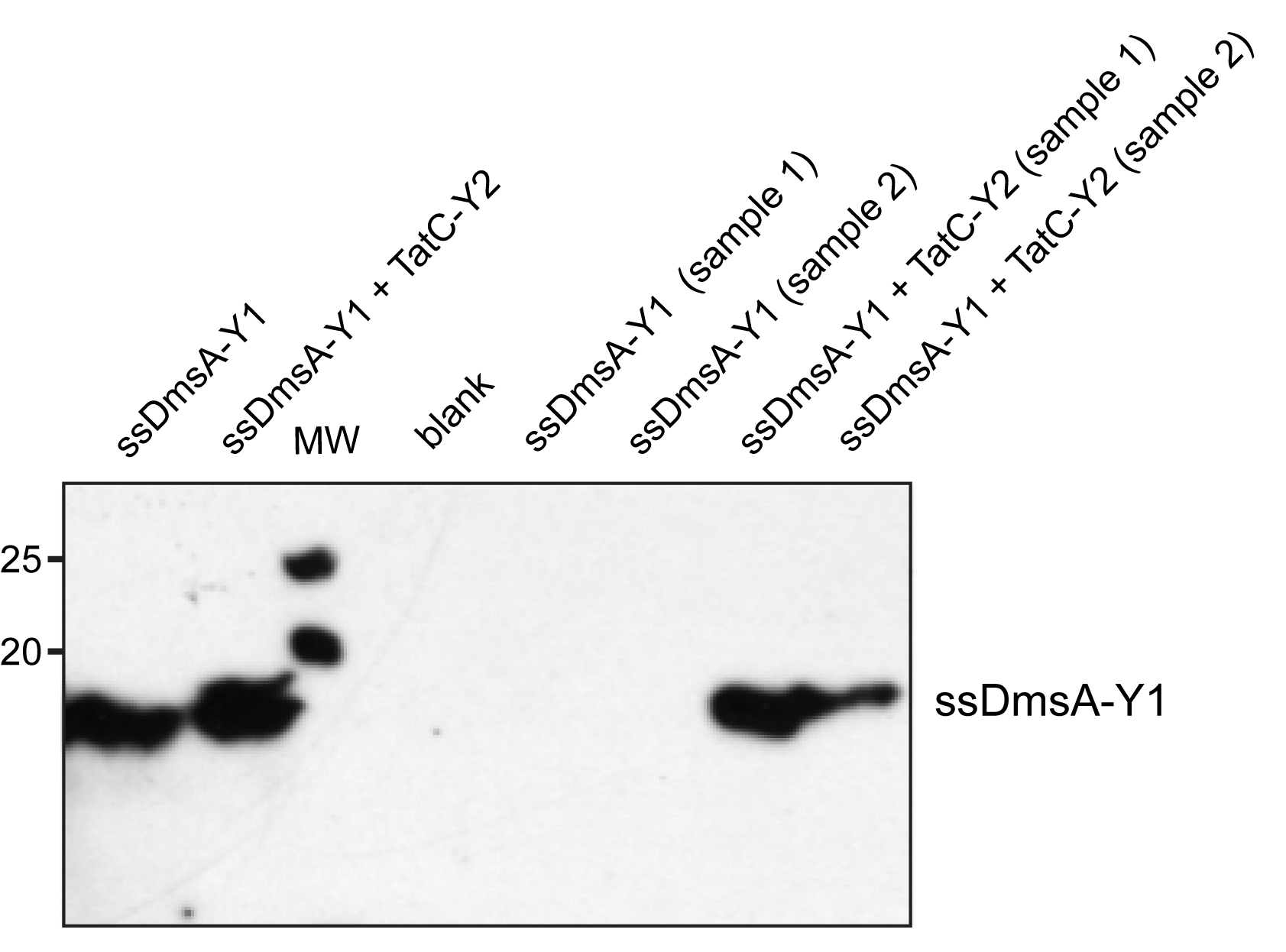

Supplement: Figure S4 — Co-localization of ssDmsA-Y1 with TatC in E. coli membranes. Western blot analysis of soluble and membrane fractions isolated from TG1 ΔtatABCE cells expressing ssDmsA-Y1 alone or co-expressing ssDmsA-Y1 with TatC-Y2. Blot was probed with anti-FLAG antibodies for detection of ssDmsA-Y1. Numbers to the left indicate the molecular weight (MW) of the ladder proteins. Two separate aliquots from the fraction collected from the top of the 70% sucrose layer (total membrane fraction) were analyzed side-by-side on the blot. An equivalent amount of soluble or membrane proteins was added to each lane. (0.58 MB TIF) [file pone.0009225.s004.tif]
