# Supplementary material for: Visualizing Interactions along the Escherichia coli Twin-Arginine Translocation Pathway Using Protein Fragment Complementation
Source: PLoS One. 2010 Feb 16;5(2):e9225. doi: 10.1371/journal.pone.0009225 (PMC2821923; doi:10.1371/journal.pone.0009225)
Supplement: Materials and Methods S1 — Text file. (0.05 MB DOC) [file pone.0009225.s005.doc]

**Supplemental Methods**

**RFP expression and analysis.** For the red fluorescent protein (RFP) BiFC assay, mRFP1 with the Q66T point mutation was split at the same amino acid location as described for YFP, generating two fragments abbreviated as R1 and R2. These fragments were cloned in place of the Y1 and Y2 fragments in the pssDmsA-Y1 and pDmsD-Y2 plasmids, respectively. For RFP fluorescence measurements, 100 μL of bacterial cells were spun down, washed twice with 1 mL of 1x PBS, and resuspended in 150 μL of 1x PBS and placed into a clear bottom-black side wall 96-well plate (Corning). Fluorescence readings were taken with a fluorescence microplate reader (Biotek Synergy HT) with excitation filter 530/25 and emission filter 590/35. Microscopy was performed on a Zeiss Axioskop 40 equipped with a Zeiss 100x/1,30 Oil Plan-NEOFLUAR lens, an X-Cite light source (EXFO, Mississauga, Ontario), a Semrock Brightline filter cube for RFP emission (Cy3-4040B-ZHE) (Rochester, NY), digitally imaged with a SPOT FLEX digital camera (Diagnostic Instruments, Inc.) and controlled with Spot Imaging Software. All images captured under 100x-oil immersion microscopy using the Zeiss 100x/1,30 Oil Plan-NEOFLUAR lens were under brightfield illumination (exposure 150 ms) or under UV illumination (exposure 500 ms).

**Substrate/chaperone co-purification analysis.** The 8xHis-DmsD-Y2 construct was co-expressed with DmsA-Y1 or ssDmsA-Y1 in TG1 Δ*tatABCE* cells (which block export of the complex) for 8 hrs at room temperature with 1mM IPTG induction in liquid LB broth. Cells were pelleted at 11,000xg for 20 min at 4^o^C. Supernatant was discarded and cell pellets were frozen at -20^o^C. Cell pellets were thawed on ice and lysed using Novagen Bug Buster Master Mix. Lysed cells were resuspended at room temperature and incubated on a rocking platform for 20 min, then spun at 11,000xg for 20 min at 4^o^C. The supernatant was passed through a 0.22 μm filter and then concentrated on a VivaSpin 20 50k MWCO filter to a final volume of 7 ml. 30 μl of 1M imidazole (5 mM final concentration) was added to the 7 ml and passed through another 0.22 μm filter before being applied to a pre-equilibrated GE HisTrap HP 1-ml column via a peristaltic pump at 1 ml/min flow rate. The flow through was collected, and the column was washed with 12 ml binding buffer (5 mM imidazole). 8-ml fractions were collected from 60-1000 mM imidazole and then concentrated to ~300 μl on 10k MWCO Millipore Amicon Ultra filters. 4 ml of storage buffer (25 mM Tris-HCl pH 8, 100 mM NaCl) was then applied for on-filter-dialysis and concentrated again to ~300 μL. For SDS-PAGE, 10 μl of concentrated protein was mixed with 10 μl of 2x SDS-PAGE buffer with β-mercaptoethanol and incubated at 100^o^C for 15 min. 10 μl of this mixture was then applied to a 4-20% SDS-PAGE gel and run at 100V for 120 min. Gels were washed in dH2O and stained with BioRad BioSafe Coomassie Blue. For native PAGE, 10 μl of concentrated protein was prepared as above but without SDS, separated on 4-20% PAGE gels, and illuminated on a UV transilluminator. Gel images were captured on a BioRad ChemiDoc.

**Membrane co-localization of DmsD with TatC.** Membrane fractions were recovered from TG1 Δ*tatABCE* cells using a sucrose density gradient. 1000-ml cultures were induced at room temperature for 8 hrs, after which cells were harvested by centrifugation at 3,200xg for 20 min using a Beckman GH-3.8 rotor. Pelleted cells were resuspended in 6 ml PBS buffer and lysed by sonication. Unbroken cells and debris were removed from lysate by centrifugation at 8,000xg for 20 min and supernatant was collected. 2 ml of supernatant was transferred on top of a two-step sucrose gradient: bottom, 4 ml of 70% (w/v) sucrose; top, 4 ml of 50% (w/v) sucrose. The gradients were centrifuged at 100,000xg for 15 h using a Beckman SW-40 rotor. The total membrane fraction was collected from the top of the 70% sucrose layer and the soluble fraction was taken from the top of 50% sucrose layer. The protein concentration of the fractions was determined using the BCA assay according to the manufacturer's instructions (Pierce). The soluble and membrane samples were adjusted to the same protein concentration prior to loading on SDS-PAGE gels.
